# Conceptus-modulated innate immune function during early pregnancy in ruminants: a review

**DOI:** 10.1590/1984-3143-AR2020-0048

**Published:** 2021-05-10

**Authors:** Cecilia Constantino Rocha, Juliano Coelho da Silveira, Niamh Forde, Mario Binelli, Guilherme Pugliesi

**Affiliations:** 1 Departamento de Reprodução Animal, Faculdade de Medicina Veterinária e Zootecnia, Universidade de São Paulo, Pirassununga, SP, Brasil; 2 Departamento de Medicina Veterinária, Faculdade de Zootecnia e Engenharia de Alimentos, Universidade de São Paulo, Pirassununga, SP, Brasil; 3 Discovery and Translational Sciences Department, School of Medicine, University of Leeds, Leeds, Yorkshire, United Kingdom; 4 Department of Animal Sciences, University of Florida, Gainesville, FL, USA

**Keywords:** Interferon-tau, Toll-like receptors, Nod-like receptors, interleukins, luteolysis

## Abstract

This review focuses on the innate immune events modulated by conceptus signaling during early pregnancy in ruminants. Interferon-tau (IFN-τ) plays a role in the recognition of pregnancy in ruminants, which involves more than the inhibition of luteolytic pulses of PGF2α to maintain corpus luteum function. For successful pregnancy establishment, the allogenic conceptus needs to prevent rejection by the female. Therefore, IFN-τ exerts paracrine and endocrine actions to regulate the innate immune system and prevent conceptus rejection. Additionally, other immune regulators work in parallel with IFN-τ, such as the pattern recognition receptors (PRR). These receptors are activated during viral and bacterial infections and in early pregnancy, but it remains unknown whether PPR expression and function are controlled by IFN-τ. Therefore, this review focuses on the main components of the innate immune response that are involved with early pregnancy and their importance to avoid conceptus rejection.

## Introduction

In cows, it is estimated that, around 10% of pregnancies do not occur due to fertilization failure, but up to 50% of pregnancies are lost in the first four weeks of embryo development due embryo/conceptus mortality (Diskin and Sreenan, 1980; Wiltbank et al., 2016). Interestingly, the largest rates of embryonic loss (60%) happen before day 17 after-mating (Reese et al., 2020). For this reason, understand events that take place in early pregnancy are a key element to preventing pregnancy loss. The major component of this is the role that the maternal immune system plays in this process (Fair, 2015).

The embryo is semi-allogenic structure containing DNA from the sire (Fair, 2015). Instead to reject this foreigner genetic material, 50% of those cows will become pregnant after the first mating/insemination (Baruselli et al., 2017). Therefore, both, the conceptus and maternal immune system must control their responses to allow pregnancy establishment. Two major components of cow’s immune system play an important role during pregnancy establishment, the adaptive (acquired immunity) and innate (natural immunity) immune systems; however, both systems biologically interact with each other during the immune response. Innate immunity has been the major component associated with immunological changes induced by early pregnancy. It utilizes receptors and cells to detect pathogens, such as Toll like receptors (TLR), complement system and natural killer cells (NK) (Hoebe et al., 2004).

During early pregnancy in ruminants, the main protein that regulates maternal recognition of pregnancy is the interferon tau (IFN-τ) (Spencer et al., 2007). IFN-τ is a glycoprotein, produced and secreted by the conceptus starting as early as day 7 of pregnancy (Lo and Summers, 2002; Sponchiado et al., 2017; Rashid et al., 2018), reaching the highest release on day 20 (Forde and Lonergan, 2017). Its main effects are to prevent the pulsatile prostaglandin F2α (PGF2α) secretion and luteolysis (Antoniazzi et al., 2013), but IFN-τ can also modulate the maternal immune response, allowing development of the semi-allogenic conceptus (Hansen et al., 2017). Therefore, this review will focus on the regulation of the innate immune system, specifically regarding the direct and indirect actions of IFN-τ during early pregnancy in ruminants.

## The IFN-τ and early pregnancy

One key factor that can modify the immune system are IFNs. In ruminants, the molecular signal that triggers maternal recognition of pregnancy is a type one interferon, IFN-τ (Roberts et al., 2008). It is detectable in the embryo as early as the blastocyst stage of development (Sponchiado et al., 2017; Talukder et al., 2017) with substantial increases starting around days 13 to 15 of pregnancy, when the conceptus is in the elongation phase (Bazer et al., 2009). The profile of IFN-τ secretion plays a key role to prevent luteolysis that would normally occur between day 15 and 18 (Spencer et al., 1995). Insufficient production of IFN-τ by the conceptus causes embryonic loss due to the inability to inhibit luteolysis (Mann and Lamming, 2001). Luteolysis is prevented by inhibition of estrogen-induced increase in estradiol receptors (*ESR1*) and oxytocin receptor (*OXTR*) expression, which are necessary to stimulate endometrial PGF2α pulses (Spencer et al., 1995; Sağsöz et al., 2011).

## Autocrine, endocrine, and paracrine actions of interferon-tau

The actions exerted by IFN-τ during early pregnancy have been studied more intensely in the last 30 years. The focus initially, was only in the paracrine effects of IFN-τ in the uterine endometrium. In contrast, the endocrine effects took place only when studies were able to detect the IFN-τ antiviral activity in the bloodstream, such as uterine vein (Schalue-Francis et al., 1991; Oliveira et al., 2008; Romero et al., 2015).

The effects of IFN-τ are exerted through a specific receptor (IFNAR) regardless the tissue type. The IFNAR is a surface receptor with two subunits (IFNAR1 and IFNAR2) (Stark et al., 1998), it regulates transcription by stimulation of signal transducers and activators of transcription (STAT) and interferon regulatory factor (IRFs) pathways (Spencer et al., 1998; Binelli et al., 2001). Once activated, the IFNAR signaling may stimulate canonical or non-canonical pathway. The canonical signaling pathway involves Janus kinase (JAK), STAT, IRF that results in interferon stimulated gene (ISG) expression. In contrast, the non-canonical pathway is not well described (Hansen et al., 2017), but it is activated by mitogen-activated protein kinase (MAPK) and phosphatidylinositol 3-kinase serine/threonine kinase 1 (PI3K-AKT1) (Stark et al., 1998).

The paracrine actions of IFN-τ in the uterine environment extend beyond the inhibition of OXTR and ESR expression. It also works through transcription of ISG such as IRF1 and IRF2 (Spencer et al., 1998), which contribute to uterine receptivity to implantation (Johnson et al., 1999; Choi et al., 2001; Henkes et al., 2015) and prevention of immune rejection of conceptus (Choi et al., 2003). Although IFNAR is expressed in the epithelium, glands, and stroma of endometrium (Rosenfeld et al., 2002), the most common ISGs are only expressed in the glandular and stromal cells (Johnson et al., 2001), excepts for MX gene, that is expressed in the luminal epithelium, but regardless of the pregnancy status (Johnson et al., 2002). Interestingly, the inhibition of ISG expression in the luminal epithelium is modulated by IRF2, as a mechanism to prevent the conceptus rejection by the immune system. (Choi et al., 2001).

Autocrine actions of IFN-τ on the conceptus are also suggested but it is not well explored as the overlap between autocrine and paracrine signal challenges to study these effects separately (Hansen et al., 2017). The idea of an autocrine effect was first generated when the IFNAR expression was described in the elongating conceptus of ewes (Wang et al., 2013). Such actions were confirmed by *in vitro* studies, based on trophoblast cell proliferation stimulated by IFN-τ in a dose-dependent manner (Wang et al., 2013). The main reason why the autocrine effects has not been well explored is the possible bias in the studies as endometrium and oviduct also contribute to conceptus development. For example, sheep lacking IFNAR in the endometrium presented malformed conceptuses (Brooks and Spencer, 2015). Additionally, day 4 embryo co-cultured with bovine oviduct epithelial cells induced production of IFN-τ in the culture media, while that did not occur in embryos cultured alone (Talukder et al., 2018). Consequently, development of *in vivo* studies to understand the isolated autocrine IFN-τ effects are not easy to be modeled but should be explored to improve our understanding of whether it plays a role in initial embryo development, endometrium, or conceptus.

An important point in IFN-τ signaling is its capability to leave the uterine lumen and acts on distant tissues in an endocrine fashion. The endocrine effects of IFN-τ were confirmed by the expression of ISG in the liver (Meyerholz et al., 2015), luteal cells (Kiyma et al., 2016; Bridi et al., 2018) and immune cells in the bloodstream (Yankey et al., 2001; Hyungchul et al., 2006; Gifford et al., 2007; Kizaki et al., 2013; Pugliesi et al., 2014). Despite the fact that, no antiviral activity was detected in the main vessels (jugular) of the circulatory system. However, in one report low levels of antiviral activity were detected in the uterine vein (Schalue-Francis et al., 1991). The IFN-τ was also detected in extracellular vesicles released by the conceptus in the circulation (Nakamura et al., 2016). Therefore, two hypotheses were generated by Hansen in 2017 (Hansen et al., 2017) to explain the mechanisms of how the IFN-τ endocrine actions occur. The first one suggests a potent action of low concentrations of IFN-τ associated with a rapid clearance from the systemic circulation, as only 58 antiviral units per mL of IFN-τ were detected in the uterine vein (Schalue-Francis et al., 1991). The second hypothesis is that IFN-τ might stimulate ISG expression through extracellular vesicles, explaining their relatively low concentrations in the peripheral circulation. The affinity of IFN-τ for IFNAR is remarkably high and it has a short half-life of 7 to 9 hours (Zhao et al., 2019). Thus, low circulating concentrations of IFN-τ could be sufficient for IFNAR stimulation (Li and Roberts, 1994).

Luteolysis is prevented by IFN-τ not only by inhibition of pulsatile PFG2α secretion, but also protecting the CL through endocrine effects (Antoniazzi et al., 2013). For example, infusion of IFN-τ into the uterine vein on day 10 of the estrous cycle in the absence of a conceptus was able to extend the CL life span in ewes and cows (Meyer et al., 1995; Bott et al., 2010). The ubiquitin like modifier 15 (*ISG15*) (Rempel et al., 2005) was detected in luteal cells of ewes on day 15 of pregnancy (Yang et al., 2010). The endocrine actions in this tissue are luteoprotective and luteotropic (Antoniazzi et al., 2013; Bridi et al., 2018). One study demonstrated that the infusion of IFN-τ into the uterine vein for 24 hours followed by an intramuscular injection of PGF2α down regulated the main membrane transporter of PGs, *SLCO2A1* (solute carrier organic anion transporter family member 2A1), and the main PGF2α receptor prostaglandin F receptor (*PTGFR*) in the CL (Banu et al., 2008; Antoniazzi et al., 2013). In addition, up regulation of prostaglandin E synthase (*PTGES*) and down regulation of cytochrome c oxidase subunit 2 (*COX-2)* in the CL occurred in response to IFN-τ. All these actions in the CL are regulated by IFNAR, mainly in the large luteal cells (Oliveira et al., 2008). Lastly, a single low dose of PGF2α (4mg/58kg) administered on day 13 in pregnant and non-pregnant ewes caused similar serum concentration of prostaglandin F metabolite (PGFM) between both, but luteolysis was only induced in non-pregnant animals (Silvia and Niswender, 1984). In non-pregnant ewes, IFN-τ alters the timing and PGFM profile in response to oxytocin treatment, and consequently prevents luteolysis induction (Ott et al., 1997). These results indicate that pregnancy does not affect the average PGFM concentrations but inhibits the pulsatile secretion of PGF2α. However, how this action is prevented still being a big question.

**Figure 1:**
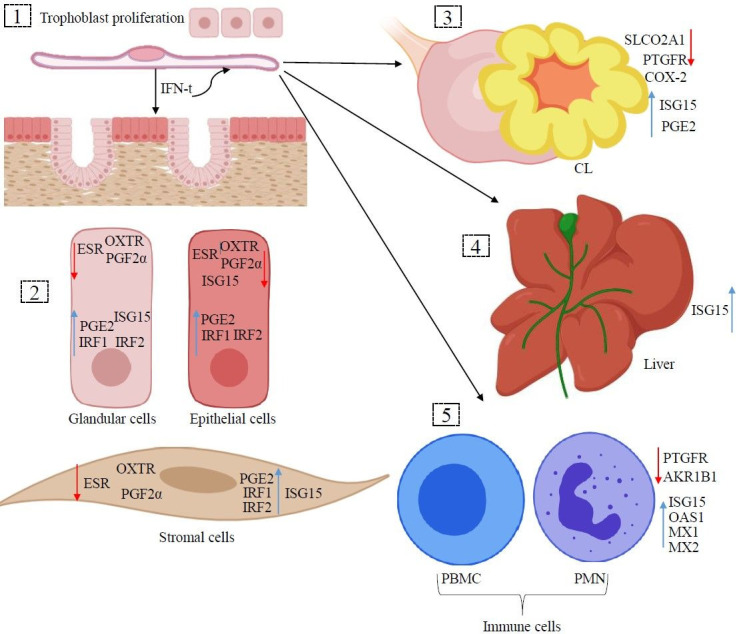
Autocrine, paracrine, and endocrine Actions of IFN-τ. The IFN-t released by the trophoblast cells (1) stimulates the conceptus development (autocrine) and (2) the transcription in the epithelial, glandular, and stromal endometrial cells (paracrine), (3) CL, (4) liver and (5) immune cells (endocrine). The effects written inside the cells represent endocrine actions, whereas outside represent paracrine actions. Red arrows represent down regulation and green arrows represent up regulation of genes.

The infusion of IFN-τ into uterine lumen induces up regulation of *ISG15* in the liver (Bott et al., 2010). Therefore, *ISG15* expression in liver and CL during pregnancy is an effect of IFN-τ stimulation, but how this endocrine effect occurs is a lack in the field. In attempt to study the endocrine effects of IFN-τ in the liver, researchers have explored ISG expression using *in vitro* studies. Cultured hepatocytes express ISG in response to IFN-τ treatment (Ruhmann et al., 2017). Consequently, this suggest that elevated ISG expression in liver biopsies of pregnant cows (Meyerholz et al., 2015) was induced directly by IFN-τ in the hepatocytes. In contrast, no function has been determined yet for the ISG regulation in those tissues. Therefore, a direct effect of IFN-τ or an indirect effect through the IFN-τ-stimulated immune cells could be involved (Ruhmann et al., 2017) and need further investigation. Endocrine, paracrine, and autocrine effects of IFN-τ during early pregnancy are illustrated on Figure 1.

**Figure 2:**
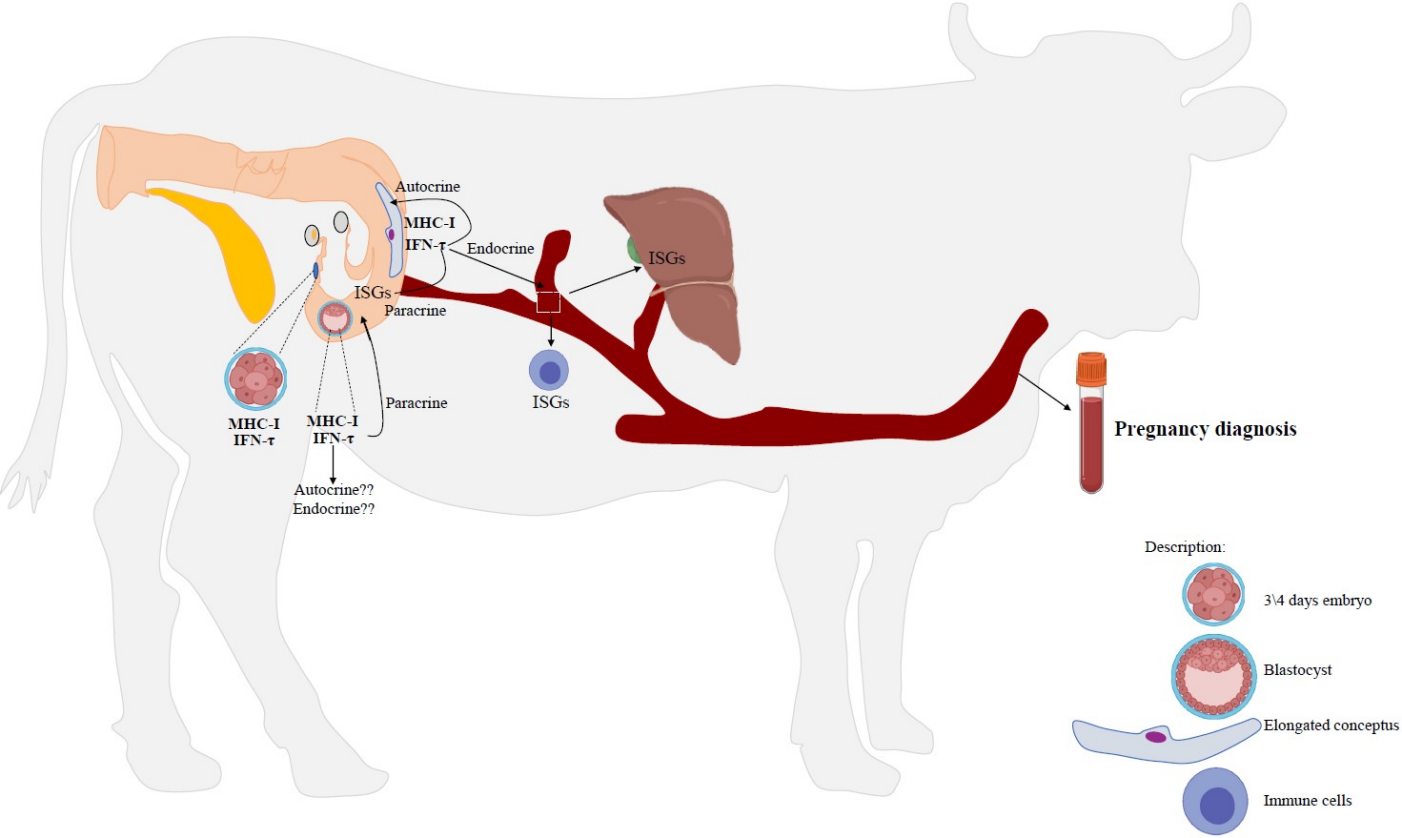
Summary of endocrine, paracrine, and autocrine actions involved in the immune system and IFN-t signaling during early pregnancy in cattle. Three steps of embryo development were represented, such as, before the blastocyst period in the oviduct environment, during the blastocyst stage in the initial part of the uterus and during the elongation phase. “??” means the absence of *in vivo* studies to prove the action. Each signal (autocrine, paracrine, and endocrine) was well detailed in the manuscript.

The expression of classic ISG in immune cells has been well studied in ruminants (Yankey et al., 2001; Hyungchul et al., 2006; Gifford et al., 2007). Independently of the cellular type, peripheral blood mononuclear cells (PBMC) or polymorphonuclear cells (PMN), the expression profile is similar with IFN-τ secretion by the trophoblast cells (Shirasuna et al., 2012; Kizaki et al., 2013; Pugliesi et al., 2014; Melo et al., 2020a). In cattle, after ISG expression reaches a peak on day 20 of pregnancy, a decrease to basal levels is observed near day 25 (Hyungchul et al., 2006; Gifford et al., 2007; Shirasuna et al., 2012; Pugliesi et al., 2014). The amount of IFN-τ released by the uterine horn correlates with the *ISG15* expression levels in the immune cells (Matsuyama et al., 2012). This agrees with the positive correlation found between ISG expression on immune cells and concentration of exogenous IFN-τ administered (Matsuyama et al., 2012). Oliveira et al. (2008) evaluated the ISG expression in whole blood from the uterine vein, uterine artery, and jugular vein, but no differences were found among the vessels in the expression of *ISG15* and 2’-5’-Oligoadenylate synthetase 1 (*OAS1*). Therefore, the endocrine effects are likely accomplished when the immune cells are exposed to IFN-τ in peripheral blood (Figure 2). The mesenteric lymph nodes also express *ISG15* (Oliveira et al., 2008) and the traffic of immune cells through that could be a source of IFN-τ stimulus. Additionally, there are *in vitro* evidence that peripheral blood mesenchymal stem cells (pbMSC) increase their migratory capacity in response to IFN-τ treatment, which could be a chemotaxis stimulus to the uterus (Calle et al., 2021).

Immune cells have important roles during pregnancy establishment (Berkowitz et al., 1988; Fair, 2015); however, the functions for each ISG up regulated in PBMC and PMN have not been determined. Infusion of PBMC from cyclic heifers into the uterine lumen (ipsilateral to the CL), on day 4 of estrous cycle, improve the quality of transferred embryos on day 7 and collected on day 15 (Ideta et al., 2010). Bloodstream immune cells on day 18 of bovine pregnancy had a down regulation of genes involved in the luteolysis process, such as *PTGFR* and aldo-keto reductase family 1 member B (A*KR1B1*) (Yang et al., 2016). These results suggest that immune cells are also involved in the maintenance of CL function.

## Immune system and early pregnancy

Two immunologic events are very important to avoid the rejection of the of the semi-allogenic conceptus during early pregnancy. Initially, at mating when the maternal reproductive tract is contacted by semen and induces the expression of immune modulators including interleukins (Ezz et al., 2019). Secondly, when the trophoblast cells begin the contact with the maternal endometrium (Fair, 2015). However, in contrast with human and mouse, research on the immune system in ruminants during early pregnancy is limited. The first studies in this field proposed three mechanisms whereby the allogenic conceptus evades the maternal immune system in vertebrates: i) conceptus being inert and not expressing histocompatibility antigens; ii) immunosuppression of the mother, and iii) the placenta being a barrier between conceptus and maternal immune cells (Medawar, 1953).

Regarding the first hypothesis, recent finds in cattle have shown that the major histocompatibility complex class I (MHC-I) genes are expressed during blastocyst development (Doyle et al., 2009) and in trophoblast cells during second half of pregnancy (Davies et al., 2000). This disagrees with the first theories suggesting an inert conceptus (Medawar, 1953). Additionally, there is a positive association between MHC-I mRNA abundance, embryo development and embryo quality in cows (Fair et al., 2004). In trophoblast cells, the non-classical monomorphic major histocompatibility complex class I (NC1) is activated by IFN-τ, IFN-gamma (IFN-γ) and high concentrations of progesterone (P4) (O'Gorman et al., 2010). The main hypothetic role of NC1 is to protect the conceptus from the NK cells attack (Ott et al., 2014). It is expressed *in vivo* in binucleate cells when they cross the maternal side (Bainbridge et al., 2001).

As cited above, immunomodulation of the mother is associated with the success of pregnancy in cattle. In ruminants, however, the number of studies in this field is low and most of them are focused on early pregnancy and IFN-τ (Ott, 2019; 2020). Basically, there is evidence that IFN-τ may modulate the immune function in the uterus by stimulating immunosuppressive molecules (Hansen, 2007). Furthermore, infusion of IFN-τ induces lymphopenia and neutropenia during ovine pregnancy (Tuo et al., 1998). When IFN-τ binds IFNAR in the endometrial tissue, beyond the classic ISG, monocyte chemoattractant protein 1 (MCP1) and MCP2 are up regulated (Mansouri-Attia et al., 2012). Both proteins are potent chemotactic factors for monocytes, which is one of the most important cell types during the immune maternal response to the embryo in cattle (Kamat et al., 2016; Velázquez et al., 2019). The maternal innate immune response is also regulated by the embryo as indicated by a down regulation of interleukin 1β (*IL1β*) and nuclear factor-ҡβ (NF-ҡβ) system (Muñoz et al., 2012). Also, early pregnancy increases the abundance of NK, cytotoxic T cells and myeloid lineage cells in the endometrium (Kamat et al., 2016; Vasudevan et al., 2017).

Another endometrial component with potential regulation by the embryo is the mesenchymal stromal cells (MSC). Researchers pointed that *in vitro* migration of endometrial MSC was reduced in front of IFN-τ treatment (Calle et al., 2019). This could be an embryo effect to retain MSC in the uterus during early pregnancy. In addition, the MSC plays role during epithelial to mesenchymal transition in the embryo implantation (Ock et al., 2016; Imakawa et al., 2018), and its immunomodulatory effect was already described through T helper cells 1 (Th1) and Th2, (Perez-Sepulveda et al., 2014; Ock et al., 2016). Therefore, have those cells in the uterine environment could be helpful to the pregnancy establishment. In front all evidence presented here, is clear that, the maternal immune system during pregnancy is not suppressed, but its immune functions in the uterus are strongly modulated (Velázquez et al., 2019).

Other important immune component strongly modulated in uterus are lymphocytes through Th cells (Maeda et al., 2013). They modulate the balance between the Th1 and Th2 responses. That response (Th1 and Th2) is balanced according to the cytokines secreted (Mosmann et al., 1986). The Th1 cells generate cytokines such as IFN-γ, IL1β and tumor necrosis factor alfa (TNFα). The Th2 cells produce IL4, IL5, IL6, IL10 and IL13 and down regulate the Th1 responses (Mosmann et al., 1986). In this sense, *in vitro* cultured trophoblast cells had a reduced growth when treated with Th1 cells (Berkowitz et al., 1988). In contrast, the cytokines such as IL12, released by the Th2 immune response induced conceptus tolerance in mice (Li et al., 1998). Therefore, the Th2 cytokines improve the success of pregnancy, while Th1 cytokines in the endometrium were unfavorable to the maintenance of pregnancy (Zhang et al., 2015). Thus, the immune control of pregnancy occurs mainly through the Th1 and Th2 response, where the Th2 is predominant. Further studies are still needed to clarify the mechanisms by which the conceptus avoids rejection during early pregnancy in cattle.

The last hypothesis proposed by Medawar was the possibility of placenta being a barrier between conceptus and maternal immune cells. This hypothesis will not be well covered in this review, as our focus is on the first three weeks of pregnancy and beginning of trophectoderm adhesion starts at the end of the third week of pregnancy in ruminants. The placenta is an organ formed by fetal and maternal tissues that mediates the exchanges between both components. Cows have a synepitheliochorial placenta; thus, the binucleated cells from the trophectoderm fuse with the epithelial tissue and likely beyond to them (Meeusen et al., 2001). In this regard, the binucleated cells begins to invade the uterine epithelium and fuse on day 19 of pregnancy in cattle. This fusion will create fetal-maternal hybrid and are particularly abundant in the placentomes. The uterus contains populations of T cells sufficient to induce graft rejection (Hansen et al., 1986); however, the proportion of lymphocytes, specially these that are present in the placentomes changes when the placentation begins. Thus, those immune cells, are reduced from the placentome on day 28 of pregnancy, close to the begin of the placentation (day 25) (Lee et al., 1997), this mechanism appears to be mediated by placenta cells to protect conceptus from the immune system attack.

## Pattern recognition receptors and type 1 interferon response

Our interest in the pattern recognition receptors (PRR) started when we performed an enrichment analysis (unpublished) with our transcriptomic data in PBMC of pregnant and non-pregnant heifers (Rocha et al., 2020). The PPR pathway came up as the most significant in PBMC of pregnant heifers on day 18 post-AI, suggesting that PRR is the main pathway affected by pregnancy during this period in immune cells.

**Figure 3:**
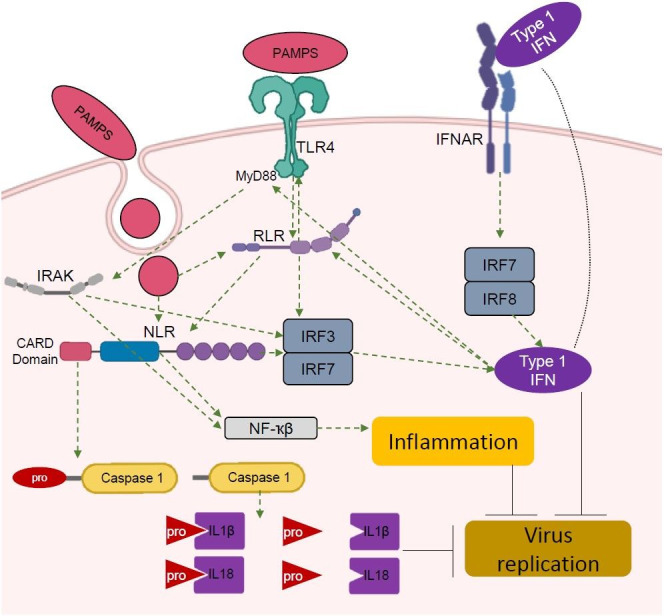
Representative synergistic collaboration among the PRR by PAMPs and type 1 IFN stimulation, where TLR4 represents the most traditional Toll like receptors. The green arrows indicate stimulation, while black strokes indicate attenuation.

The PRR pathway is involved in the innate immune system and plays a role to identify pathogens by specifically engaging pathogen-associated molecular patterns (PAMPs) of each antigen (Thompson et al., 2011) (Figure 3). The most studied PRR are the TLRs, which are type 1 transmembrane proteins responsible for the detection of PAMPs such as, bacterial lipopolysaccharides (LPS) and viral nucleic acids in the extracellular environment (Thompson et al., 2011). In addition, nucleic acids from viruses have emerged as major components of the TLRs response, and more particularly, toll like receptor 4 (TLR4) is an essential member of the TLRs family (Dai et al., 2016). The TLR4 receptor has an intracellular subdomain, the myeloid differentiation factor 88 (MyD88) (Balloy et al., 2008). The Myd88 domain activates the TLR4 when the type 1 interferon (IFN) binds a sub protein in its structure, the toll/interleukin 1 receptor (TIR) domain (Dai et al., 2016) (Figure 3).

In the cytoplasm, there are other PRR components involved in the immune response, like retinoic acid-inducible gene I-like receptors (RLRs) and Nod like receptors (NLRs) (Thompson et al., 2011). When the PRR pathway is activated, the main result is the transcription of essential factors, such as IRFs (IRF3, 7 and 8), cytokines and maturation of IL1β and IL18 (Figure 3). As the PRR play a role in the viral response, they are also regulated by type 1 IFNs. The mRNA of several TLRs (*TLR2*, *TLR4*, *TLR5*, *TLR6*, *TLR7*, *TLR8*, and *TLR10*) were detected in the ovine trophoblast on day 13 post mating (Kaya et al., 2017), but not in the immune cells. Furthermore, IRFs are the key mediator of TLRs immune response and thus, *IRF7* and 6 were found in the exosomes released by trophoblast cells in the uterine lumen of ewes from days 13 to 16, with the highest expression on day 14 after mating (Ruiz-Gonzalez et al., 2015).

There is a synergistical relationship among PRR (Oviedo-Boyso et al., 2014). For example, NLRs are activated intracellularly by TLRs and RLRs (Kawai and Akira, 2011) (Figure 3). In this regard, RLRs act as a cytoplasmic sensor of viral RNA and type I IFN production. The activated RLRs activate NF-ҡβ and IRFs, both crucial to the induction of type I IFN (Loo and Gale, 2011) (Figure 3). In addition, type 1 IFN activate RLRs in dendritic cells, which consequently activate TLRs (Loo and Gale, 2011). The TLRs may also stimulate the phosphor-interleukin-1 receptor associated kinase (IRAK), which contributes to interleukin production in the immune response (Wang et al., 2017). In this context, there is a member of the RLR family, termed retinoic acid inducible gene 1 (RIG-1), which is an enzyme encoded by the DExD/H-box helicase 58 (*DDX58*) gene (Wu et al., 2019). Interestingly, DDX58 is an ISG up regulated in the endometrium on day 16 of pregnant cattle (Forde and Lonergan, 2012). Additionally, when IFN binds its receptor, two kinds of transcription events are activated, the fast response (~2 hours) and the slow response (~24 hours) (Forde et al., 2012). Regarding PRR there is a human model where TLRs mediate the fast response of IFN while the RLR participate of the slow phase (Agod et al., 2017). Lastly, TLR2/4 agonists are able to induce the PGE_2_ synthesis in endometrial explants (Li et al., 2020).

The NLRs constitute a large family of intracellular receptors, also stimulated by IFN (Fekete et al., 2018) in the PRR group. They can be classified in three subgroups: inflammasome (NLRP1, NLRP3, NLRP6, NLRC4 and NLRC5), reproductive NLRs (not classified yet) and regulatory NLRs (NOD1, NOD2, NLRP12, NLRX1 and NLRC3) (Coutermarsh-Ott et al., 2016). They are characterized by a tripartite domain structure with a N-terminal domain that typically includes either Pyrin or Caspase recruitment (Card) domains, a central nucleotide, and a C-terminal domain (Schroder and Tschopp, 2010). The NLRs modulate important signaling pathways such as NF-ҡβ and IFN responses (Coutermarsh-Ott et al., 2016). Interestingly, as the NLRs, TLRs and RLRs are interlinked. When the PRR are activated, the outcome will be activation or formation of multiprotein complexes that have the capacity to modulate inflammation or the immune response (Figure 3). Therefore, some consequences of activation include ILs and caspase 1 (CASP1) maturation, IRFs up-regulation, and ISG expression including *ISG15* and MX dynamin like GTPase 1(*MX1*) (Wei et al., 2016). Modulation of NLRs in reproduction was first described in human oocytes and ovaries in a meta-analysis (Hruz et al., 2008). In this regard, NLRP7, 5 and 9 proteins are expressed during human follicle development (Sena et al., 2009). The transcripts *TLR2* and *TLR4* are expressed in the uterine luminal and glandular epithelial cells (Ezz et al., 2019). Involvement of TLRs in the uterus appears to be linked with immune response to the sperm through activation of PRR pathway (Ezz et al., 2019). Besides the activation of the pathway and overlap among PRR and IFN-τ-induced transcripts, no specific role for PRR in bovine reproduction was explained yet.

## Understanding the conceptus-modulated immune system for applied purposes

Considering that the ISG expression profile in circulating immune cells follows the same of profile of IFN-τ secretion (Yankey et al., 2001; Hyungchul et al., 2006; Gifford et al., 2007), several research groups (Stevenson et al., 2007; Green et al., 2010; Kizaki et al., 2013; Pugliesi et al., 2014) have tried to develop an earlier pregnancy diagnostic test in cattle. Most of the studies identified days 18 to 20 of pregnancy as the best period for comparison of *ISG15*, *MX1*, *MX2* and *OAS1* expression between pregnant and non-pregnant cattle (Hyungchul et al., 2006; Gifford et al., 2007; Green et al., 2010; Shirasuna et al., 2012; Pugliesi et al., 2014). This profile is conserved among the studies, independent of the parity status, breed, and embryo origin (AI or embryo transfer) (Matsuyama et al., 2012).

Expression of ISG in immune cells was tested as a prospective diagnostic method to detect pregnancy on day 20 in heifers and cows. It resulted in 62% to 80% of accuracy regardless the cell type (PBMC or PMN) (Pugliesi et al., 2014; Melo et al., 2020b). Therefore, to improve the accuracy of this methodology for pregnancy prediction, ISG were measured at two time points, one at the time of AI and second on day 18 post-AI (Green et al., 2010). The resultant relative expression of each animal from both days is used to calculate a ratio value. Where, a cut-off value of the expression ratio between day 18 and day 0 (time of AI) is defined to classify the animal as pregnant or non-pregnant. This approach has shown higher accuracy when compared to the single expression evaluated on day 20 (Green et al., 2010).

The inaccuracy to predict the pregnancy status through ISG is a result in part of a great proportion of false-positive results and could be a consequence of an early pregnancy loss or induction of ISG by infection or other stimulus that induce IFN expression. IFNAR is a non-selective receptor and is stimulated by any type 1 IFN, which means that other IFNs could bind it and stimulate ISG expression like in viral infections (Takino et al., 2016). In this same way, most embryo losses happen before day 18 of pregnancy (Diskin and Sreenan, 1980; Wiltbank et al., 2016), but an earlier secretion of IFN-τ could have occurred in animals that underwent embryo loss, increasing the false positive rate. When pregnant, non-pregnant and embryo death (by PGF2α on day 18 of pregnancy) were evaluated in ewes, no difference was found for embryo death and pregnant ewes on ISG expression on day 21 and 23 post-mating (Kose et al., 2016). Another important point to be considered is the false negative rate, which must be null for a pregnancy diagnosis. When the ISG expression was used for early pregnancy diagnosis (day 20) the false negative rate reached up to 30% (Melo et al., 2020b). For this reason, 30% of the animals would be considered non-pregnant when in fact were pregnant. The false negative results may be associated to animals that the IFN-τ does not signal enough to stimulate the ISG expression. In this regard, it is known that the conceptus size is directly correlated with the amount of IFN-τ released (Hernandez-Ledezma et al., 1993).

## Conclusions

This review discussed some gaps in our knowledge regarding immune modulation during early pregnancy. The autocrine actions IFN-τ (Yankey et al., 2001; Hyungchul et al., 2006) is a recent find and should be better understood. After all these studies in the last 30 years, we are not able to differentiate which actions involved in the embryo development are being stimulated from either, itself or the uterus. For this, *in vitro* and *in vivo* studies using knockout cell could be an interesting alternative and solve this fundamental query. The involvement of the innate immune system in paracrine and endocrine actions of IFN-τ and other conceptus signals need to be explored. The role of the innate immune system and PRR in establishment of pregnancy is not known. The adaptive immune system also should be investigated, especially, because there are differences in immune response between cows and heifers (Melo et al., 2020b). Cows that already experienced a pregnancy could have a smother adaptative immune response than compared to heifers, that never had this experience before. For this, culture of specific immune cells treated with molecules present during early pregnancy could be helpful allowing a better comprehension of the events involved in early pregnancy and its losses. Finally, the use of ISG to predict pregnancy establishment, or, more importantly, failed or lack of pregnancy, is an interesting and innovative approach for cattle industry, but much work still needs to be done. Thus, the better understanding of the basic events involved in modulation of immune system during early pregnancy is the first step for determining new markers and endpoints for prediction of pregnancy earlier than the traditional methodologies.
